# Preventive therapy with galcanezumab for two consecutive cluster bouts in patients with episodic cluster headache: an observational multicenter study

**DOI:** 10.1186/s10194-023-01661-7

**Published:** 2023-10-10

**Authors:** Yooha Hong, Mi-Kyoung Kang, Heui-Soo Moon, Byung-Kun Kim, Soo-Jin Cho

**Affiliations:** 1 Department of Neurology, Dongtan Sacred Heart Hospital, Hallym University College of Medicine, Keun Jae Bong-gil 7, Hwaseong, Gyeonggi-do 18450 Republic of Korea; 2 Department of Neurology, Kangbuk Samsung Hospital, Sungkyunkwan University School of Medicine, Seoul, Korea; 3 Departement of Neurology, Nowon Eulji Medical Center, Eulji University School of Medicine, Seoul, 01830 Republic of Korea

**Keywords:** Cluster headache, Cluster bout, Calcitonin gene-related peptide, Episodic, Galcanezumab, Preventive treatment

## Abstract

**Background:**

Cluster headache is a severe and disabling primary headache disorder. Galcanezumab is a monoclonal antibody against calcitonin gene-related peptide and a preventive therapy for episodic cluster headache. However, the approval and insurance coverage for episodic cluster headache differ in each country. Additionally, the consistency of efficacy of galcanezumab therapy has not yet been evaluated. This study aimed to assess the efficacy and safety of 240 mg of galcanezumab therapy for consecutive cluster bouts in patients with episodic cluster headache.

**Methods:**

The study enrolled patients with episodic cluster headache who received two courses of galcanezumab therapy at three university hospitals in Republic of Korea between February 2020 and April 2022. The efficacy and safety of galcanezumab were analyzed by comparing daily headache frequency, the number of headache days, and headache intensity and adverse effects during the one-week period before and the third week after galcanezumab injection for each episode of cluster bouts. Paired t-test was used for comparing repeated data from different episodes of cluster bout.

**Results:**

Sixteen patients were enrolled in this study. Fourteen patients received galcanezumab therapy for two consecutive cluster bouts. Galcanezumab was administered 24 days and 11 days after the first and second cluster bouts, respectively. The proportion of patients with 50% or more reduction in frequency of daily headache at week 3 from baseline was 86% and 64% during the first and second episodes, respectively. The proportion of patients who received transitional therapy before galcanezumab therapy was higher in the first episode of cluster bout than that in the second episode of cluster bout. No serious adverse reactions or significant differences in adverse effects between cluster bouts were noticed. Two patients received a second galcanezumab therapy during the pre-cluster period, and their cluster periods ended without typical cluster headache attacks 10–60 days after galcanezumab therapy.

**Conclusions:**

This exploratory analysis suggests that galcanezumab may be effective as a preventive therapy in subsequent cluster bouts. Patients with episodic cluster headaches who underwent galcanezumab therapy tended to receive a second round of treatment in the early stages of their next cluster bout without transitional therapy.

## Background

Cluster headache (CH) is a primary headache disorder characterized by disabled trigeminal autonomic cephalalgia and severe unilateral headache lasting for 15–180 min [1]. The pain of CH attack is more severe than any other painful conditions and rated 9.7 out of 10 on the numerical rating scale [2]. This significant pain has a substantial impact on the patient’s quality of life and, in some cases, leads to suicidal thoughts or attempts [1, 3]. The recent study from European academy published the guidelines on treatment of CH and introduced several acute and prophylactic managements [4].

Galcanezumab, a monoclonal antibody against calcitonin gene-related peptide, is one of the novel prophylactic treatments of CH. It has received approval for episodic CH from the Food and Drug Administration in the USA but has not been approved in Europe [5]. Subcutaneous administration of three 100 mg syringes, at a total dose of 300 mg galcanezumab per month during the cluster period results in the proportion of patients with 50% or more reduction in weekly CH attacks at week 3 from baseline by 78.8% [6]. However, a 300 mg dose is unavailable in many countries, including Korea. Therefore, a 240 mg (two prefilled syringes of 120 mg each) dose of galcanezumab followed by another 120–240 mg monthly is used as an alternative for patients with CH [7–11]. The safety and satisfaction in a long-term open-label study of consecutive galcanezumab treatment have been favorable in patients with CH [12], however, clinical practice in headache clinic of galcanezumab treatment as case series in patients with episodic CH are still limited. The efficacy of galcanezumab treatment for subsequent cluster bouts has not yet been evaluated in patients with CH.

This study aimed to investigate the preventive efficacy and safety of 240 mg of galcanezumab treatment for consecutive cluster bouts in patients with episodic CH in headache clinical practice. Our hypothesis was that patients would administer injection earlier during the subsequent cluster bouts, potentially resulting in greater effectiveness in real-world practice.

## Methods

### Study population

**Fig. 1:**
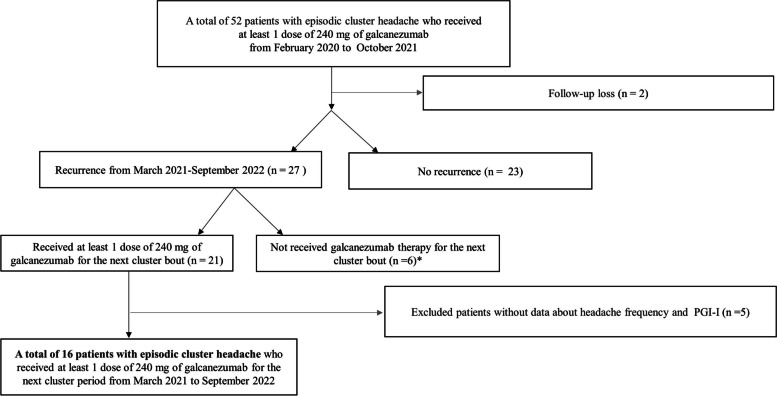
Selection of patients with cluster headache. PGI-I; Patients Global impression of improvement. * Causes of not receiving galcanezumab therapy for their cluster bout were poor efficacy or prefer other treatment in 5 patients and economic burden in 1 patient

This observational and multicenter study included patients with CH, who received at least one dose of 240 mg galcanezumab (two prefilled syringes of 120 mg each) in different episodes of cluster bouts at three university hospitals from February 2020 to October 2022. The patients had a history of CH, as defined according to the diagnostic criteria of the third edition of International Classification of Headache Disorders (ICHD-3) [13]. Three neurologists evaluated the patients and diagnosed CH based on patient’s history and clinical presentation using the ICHD-3. The study protocols for the prospective and retrospective registries were approved by the institutional review board of each hospital (EMCS 2021–10–032–001). For patients who received galcanezumab treatment to prevent CH before the institutional review board approval, the IRB waived the requirement for written informed consent owing to retrospective data collection and full anonymity. After IRB approval, all patients were informed in detail about the study purpose, and they provided written informed consent before voluntary participation. Of a total of 16 patients, 10 were included in retrospective registration and 6 were included in prospective registration. The study was conducted in accordance with the principles of the Declaration of Helsinki. The inclusion criteria for participants were as follows: 1) patients diagnosed with CH, 2) 19–60 years of age, and 3) patients who received at least one galcanezumab dose for different episodes of cluster bout. We excluded patients who did not record their headache frequency or Patient Global Impression of improvement (PGI-I) in their headache diary. The participant flowchart is shown in Fig. 1.

### Assessment of cluster headache

All patients completed a structured questionnaire designed to evaluate CH in the Korean Cluster Headache Registry (KCHR). The KCHR protocol evaluates sociodemographic variables, including sex, age at the onset and presentation, body mass index (BMI), and history of smoking and alcohol consumption. Additionally, we collected baseline characteristics of CH, including age at onset, duration of CH, and mood change (depression, anxiety, and suicidal thoughts). Patients from the prospective registry were asked to maintain a headache diary and other structured questionnaires for further evaluation of CH during galcanezumab treatment. We investigated the features of CH, including the duration between each episode of cluster bouts, pre-attack symptoms and pre-cluster symptoms, injection timing, acute and preventive treatments, headache frequency of CH attacks, number of days with acute medication and severity of pain.

Based on the previous studies [14–16], we described pre-attack symptoms as the signs and symptoms that precede before CH attacks, indicating an imminent onset in the patient. And pre-cluster symptoms were defined as the presence of CH-related symptoms occurring days or weeks before the onset of the cluster bouts. To assess mood changes, we used the Patient Health Questionnaire-9 (PHQ-9), a Generalized Anxiety Disorder (GAD)-7, and Euro-Quality of Life-5 Dimension (EQ-5D). A total score of ≥ 10 on the PHQ-9 indicated the presence of a significant depressed mood [17]. Anxiety was indicated by a GAD-7 and a total score of ≥ 6 indicated the presence of anxiety [18]. The EQ-5D is a commonly used tool for evaluating health status and quality of life. It assesses health status across five dimensions (mobility, self-care, usual activities, pain/discomfort, anxiety/depression) and adjusts the level of each dimension to measure health-related quality of life [19]. And we checked whether the patient had ever thought of suicide due to CH.

### Assessment of efficacy and safety of galcanezumab therapy

The efficacy and safety of galcanezumab were analyzed in patients with CH according to each episode of cluster bout. The primary objective was to assess a 50% decrease in daily headache frequency (number of headache attacks per day). Additionally, the reduction in the number of headache days and headache intensities were evaluated one week before and three weeks after administrating galcanezumab for each episode of cluster bouts. The evaluation of headache intensity was performed using Visual Analog Scale (VAS) scores, which range from 0 to 10. PGI-I and adverse drug reaction were assessed separately for each cluster bouts. PGI-I is used to measure the severity of symptoms and treatment efficacy in a treatment process or study. Patients’ responses were rated from 1 (very much better) to 7 (very much worse) on the PGI-I scale [5, 20]. Safety assessment data, including adverse drug reaction, were collected from patients’ medical records, self-reported headache diaries, or telephone interviews.

### Statistical analysis

Categorical data are presented as frequencies and percentages (%). Age, onset age and BMI are presented as mean and standard deviation. The remaining continuous dataare presented as medians and quartiles (interquartile range). For comparison of repeated data according to different episodes of cluster bout, a paired *t*-test was used for continuous or categorized variables. Nonparametric tests were used to establish statistical significance at *p* < 0.05 when the normality assumption was not met. Data were analyzed using SPSS v.24 (SPSS, Chicago, IL, USA).

## Results

### Baseline characteristics of the enrolled patients with episodic cluster headache

During the study period, out of the fifty-two patients with episodic CH who were administered 240 mg of galcanezumab, twenty-seven experienced a relapse. And twenty-one patients who received 240 mg of galcanezumab again were included in the study. After excluding five patients according to criteria, sixteen patients with episodic CH, who received at least one dose of 240 mg galcanezumab, experienced recurrent cluster bout and were enrolled in this study. Of these, fourteen patients (87.5%) received at least one dose of 240 mg galcanezumab (two prefilled syringes each containing 120 mg) in consecutive episodes of cluster bouts, and the remaining two patients received a second treatment with galcanezumab during the pre-cluster period.

In the 14 patients who received galcanezumab treatment in two consecutive cluster bouts, the mean age was 38.1 ± 10.2 years. The mean onset age of CH was 26.4 ± 12.9 years and the median duration of CH disease before the 1^st^ galcanezumab treatment was 3.1 (interquartile range, IQR 0.6,8.9) years (Table 1). Three patients (21%) were current smokers and five (36%) had a previous history of migraines. The average scores of PHQ-9 and GAD-7 were 12 and 10, respectively. Aspects of quality of life, the median EQ-5D scores was 0.9 (IQR, 0.7, 0.9), and no patient attempted suicide.

**Table 1:** Baseline characteristics of the 14 patients with episodic CH who received GT in different cluster bouts

Age, years	38.1 ± 10.2
Male sex, n (%)	9 (64.3)
Onset age, years	26.4 ± 12.9
Duration of CH disease before 1^st^ GT, years	3.1 (0.6, 8.9)
Duration between 1^st^ GT and 2^nd^ GT, months	10.0 (6.5, 17.5)
BMI, kg/m^2^	23.8 ± 2.5
Ever-smoker, n (%)	3 (21.4)
Current alcohol drinking, n (%)	5 (35.7)
Comorbid migraine, n (%)	5 (35.7)
PHQ-9 score^a^	12.0 (5.0, 17.0)
GAD-7 score^a^	10.0 (2.5, 15.0)
EQ-5D scores^a^	0.9 (0.7, 0.9)
Passive suicidal idea^a^	3 (21.4)

Age, onset age and BMI are presented as mean ± standard deviation. The remaining data are presented as median (25^th^ to 75^th^ quartile) according to normality of variable

*CH* Cluster headache, *GT* Galcanezumab therapy, *BMI* Body mass index, *PHQ-9* Patient Health Questionnaire, *GAD-7* Generalized Anxiety Disorder, *EQ-5D* Euro-Quality of Life-5 Dimension

^a^ Data about psychiatric comorbidities and suicidal idea were available among 11 patients. No patient attempted suicide

### The timing and kinds of preventive treatments in two consecutive episodes of cluster bouts

The median duration between 1^st^ and 2^nd^ galcanezumab treatments was 10.0 (IQR, 6.5, 17.5) months. The median duration of cluster bout period was 38.0 days in the first episode and 37.5 days in the second episode, respectively (Table 2). Galcanezumab was injected median 24.0 (IQR, 14.3, 34.3) days after the onset of the first episode of cluster bout and 11.0 (IQR, 5.0, 26.5) days in second episode (95% confidence interval [CI] -0.8 to 15.8, *p* = 0.071).

**Table 2:** The timing and kinds of preventive treatments in two consecutive episodes of cluster bouts (*N* = 14)

Characteristics	1^st^ GT-treated episode	2^nd^ GT-treated episode	95% CI	*P-*value
Total duration of cluster bout, days	38.0 (25.3,65.8)	37.5 (16.3,77.0)	[-19.6, 23.3]	0.855
Time to GT from the onset of cluster bout, days	24.0 (14.3,34.3)	11.0 (5.0–26.5)	[-0.8, 15.8]	0.071
Time to end of cluster bout following GT, days	12.0 (2.5,48.3)	17.5 (8.5–43.3)	[-17.5,14.9]	0.867
**Preventives before GT**				
Transitional preventives	4 (28.6)	0 (0.0)		0.049†
Prednisolone	4 (28.6)	0 (0.0)		0.049†
GONB	3 (21.4)	0 (0.0)		0.111
Conventional Preventives	5 (35.7)	2 (14.3)		0.272
Verapamil	4 (28.6)	2 (14.3)		0.324†
Lithium	1 (7.1)	0 (0.0)		0.500†
Topiramate	3 (21.4)	0 (0.0)		0.111
**Additional preventives after GT**				
Transitional preventives	4 (28.6)	7 (50.0)		0.220
Prednisolone	3 (21.4)	5 (35.7)		0.339†
GONB	2 (14.3)	5 (35.7)		0.192†
Conventional Preventives	8 (57.1)	10 (71.4)		0.336
Verapamil	7 (50.0)	6 (42.9)		0.500
Lithium	4 (28.6)	2 (14.3)		0.324†
Topiramate	2 (14.3)	2 (14.3)		0.702†

Data presented as are presented as median (25^th^ to 75^th^ quartile) or number (%). These *p*-values are based on paired-t test or chi-square test

*GT* Galcanezumab therapy, *CI* Confidence Interval, *GONB* Greater occipital nerve block*

^†^ Fisher’s Exact Test

The numbers of patients who received galcanezumab treatment as the only preventives were 5 patients in the first episode and 4 patients in the second episode (*p* = 0.336). Regarding total preventives used during each episode of cluster bout, there were no differences in transitional therapy (50% in each episode) or conventional preventives (36% in each episode). Two patients during the first episode of cluster bout and 5 patients during the second episode of cluster bout received the follow-up galcanezumab treatment of 120 or 240 mg during the second month of cluster bout.

Regarding preventives before galcanezumab treatment, four patients received transitional therapy in the first episode and none of the patients received transitional therapy in the second episode of cluster bout (29% vs. 0%, *p* = 0.049). There were no differences in the proportion of each preventive therapy before the two episodes of cluster bout (36% vs.14%, *p* = 0.272). Regarding additional preventives treated after galcanezumab treatment, there were no differences in the proportions of patients receiving transitional (29% vs.50%, *p* = 0.272) and conventional (57% vs.71%, *p* = 0.336) preventives during each of the two episodes of cluster bout.

The proportions of patients with 50% or more reduction in daily headache frequency at week 3 from baseline were 86% and 64% during the first and second episodes of cluster bout (*p* = 0.192). Treatment responses, PGI-I, and adverse drug reaction were generally similar between the two episodes of cluster bout (Table 3). In the 1^st^ episode of galcanezumab treatment, a decrease of five headache days in week 3 after galcanezumab treatment was noticed compared to that one week before treatment. In the 2^nd^ episode of galcanezumab treatment, a decrease of three headache days at week 3 after galcanezumab treatment was observed during the same period (*p* = 0.004). No significant differences in the changes of daily headache frequency (-1.0 vs. -0.9, *p* = 0.965), total number of headaches (-7.0 vs.-6.0, *p* = 0.975), and VAS scores (-7.5 vs. -5.0, *p* = 0.251) were observed between the two galcanezumab treatment episodes. The improvement of PGI of galcanezumab treatment was reported as feeling “very much better” or “much better” in 86% and 64% patients in the first and second episodes, respectively. No serious adverse reactions or statistically significant differences in the frequency of adverse events according to the episodes of cluster bout were noticed.

**Table 3:** The clinical characteristics according to episode of cluster bout treated GT (*N* = 14)

	1st GT treated episode		95% CI	*P*	2nd GT treated episode		95% CI	*P*
	1 week before GT	3 weeks after GT			1 week before GT	3 weeks after GT		
Headache characters								
Headache days	7.0 (5.0,7.0)	0 (0, 2.5)	[3.4, 6.2]	< 0.001*	4.0 (4.0, 7.0)	2.0 (0.0, 7.0)	[0.1, 4.2]	0.041*
Daily headache frequency	1.0 (1.0,1.6)	0 (0,0.6)	[0.6, 1.5]	< 0.001*	1.0 (0.9, 2.2)	0.4 (0.0, 1.0)	[0.2, 1.9]	0.021*
Number of total headaches	7.0 (7.0,11.8)	0 (0,4.0)	[4.3, 10.4]	< 0.001*	7.0 (6.5, 15.5)	2.0 (0.0, 7.8)	[1.4, 13.4]	0.019*
Headache intensity (VAS)	8.0 (7.0,9.0)	0.0 (0.0,3.3)	[3.8, 8.2]	< 0.001*	8.0 (7.8,9.0)	3.0 (0.0,5.0)	[3.6, 6.5]	< 0.001*
3 weeks after—1 week before								*P*
Headache days	– 5.0 (–7.0, –2.8)				–3.0 (–4.0, 0.5)		[-4.3, -1.0]	0.004*
Daily headache frequency	– 1.0 (–1.4, –0.6)				–0.9 (–1.8,0.0)		[-3.0, 4.3]	0.965
Number of total headaches	– 7.0 (–10.3, –4.5)				–6.0 (–12.5, –1.5)		[-6.0, 3.6]	0.975
Headache intensity (VAS)	– 7.5 (–9.0, –2.3)				– 5.0 (–7.3, –2.8)		[-3.5, 1.0]	0.251
PGI-I, n (%) ^a^								
Very much better			8 (57.1)		3 (21.4)			0.300
Much better			4 (28.6)		6 (42.9)			
A little better			1 (7.1)		3 (21.4)			
No change			1 (7.1)		2 (14.3)			
ADR, n (%)	4 (28.6)				3 (21.4)			0.671
Skin rash	0 (0.0)				2(14.3)			0.481
Constipation	4 (28.5)				1 (7.1)			0.326

Data presented as median (25th to 75th quartile) or number (%). These *p*-values are based on paired t-test or chi-square test

*GT* Galcanezumab therapy, *CI* Confidence Interval, *VAS* Visual Analog Scale, *PGI-I* Patient Global Impression of improvement, Clinical Global Impression, *ADR* Adverse Drug Reaction

* *p*-value<0.05, ^a^ Fisher's Exact Test

### Two cases receiving galcanezumab treatment during the pre-cluster period

Two patients received the 2^nd^ galcanezumab treatment during the pre-cluster period and did not experience cluster bout thereafter, and duration of the pre-cluster period was 60 days in case 1 and 10 days in case 2.

In case 1 (a 48-year-old, male), before the 1^st^ galcanezumab treatment, the duration of cluster bout was usually 12 weeks. During the 1^st^ galcanezumab treatment period, duration of the first cluster bout episode was 60 days after five days of the pre-cluster period, with a feeling of the upcoming cluster bout. After 11 months, the patient experienced the same pre-cluster symptoms for 14 days and received the 2^nd^ galcanezumab treatment. The pre-cluster symptoms ended without typical CH 47 days after the 2^nd^ galcanezumab treatment.

In case 2 (a 32-year-old, female), the usual duration of cluster bout was 11 weeks before the 1^st^ galcanezumab treatment. She received 1^st^ galcanezumab treatment 29 days after the onset of cluster bout, which ended 46 days after the 1^st^ galcanezumab treatment. After 10 months, the patient experienced pre-cluster symptoms with a feeling of the upcoming cluster bout of very mild pain for eight days. She received the 2^nd^ galcanezumab treatment, and her pre-cluster symptoms disappeared two days after the 2^nd^ galcanezumab treatment without cluster bout.

## Discussion

Our results suggested that 240 mg of galcanezumab can be effective for patients with episodic cluster headache in consecutive cluster bouts. This is the primary study to explore patients with intermittent CH in actual clinical practice at a headache clinic using data from the CH registry. The patients with episodic CH, who had experienced galcanezumab treatment tended to receive a second galcanezumab treatment during early stages of the next cluster bout (11 days vs. 24 days). Probably, the positive experience of the 1^st^ galcanezumab treatment encouraged the patients to have another galcanezumab treatment at the beginning of the next cluster bout. Additionally, 240 mg galcanezumab treatment to prevent CH was effective for each cluster bout in this study. The proportions of patients with a 50% or more reduction in the headache frequency per day at week 3 from baseline were 86% and 64% during the first and second episodes of cluster bout. This difference may be caused by differences in the timing of galcanezumab treatment, high proportion of transitional therapy before 1^st^ galcanezumab treatment, and selection bias of patients who wanted to receive 2^nd^ galcanezumab treatment. In the 1^st^ galcanezumab treatment, the timing of injection was delayed; therefore, some patients might receive it at the end stages of cluster bout. Therefore, the efficacy of galcanezumab treatment of 240 mg might be more accurately evaluated during the second cluster bout.

The most appropriate timing for galcanezumab treatment is uncertain for the cluster period. In relation to the predictability and prevalence of pre-cluster symptoms [14, 15], several studies have reported that approximately 35–86% patients can predict upcoming cluster bout several days before the onset [21]. If the onset of cluster bouts can be predicted in advance and pre-treatment is administered, the management of CH pain can become more effective and beneficial. Particularly, recognition of pre-cluster symptoms can help prevent the onset of cluster attacks by facilitating the early initiation of preventive therapy. However, proper management of pre-cluster symptoms remains unsolved. In this study, two patients received galcanezumab treatment during the pre-cluster period and experienced pre-cluster symptoms only without a full-blown cluster bout. However, it’s worth noting that even in these cases, the pre-cluster period was not consistently observed prior to every cluster bout. This variability poses a significant clinical challenge, as patients may undergo preemptive treatments based on the presence of pre-cluster symptoms, only to find that a full cluster bout does not always follow. These findings underscore the complexity of the relationship between pre-cluster symptoms and the actual occurrence of cluster bouts. Future research efforts aimed at deciphering the factors contributing to the transition from pre-cluster symptoms to an actual bout are crucial to tailor interventions more effectively.

And treatment for CH consists of short-acting abortive therapy, preventive therapy and transitional therapy that effectively halts ongoing attacks [22]. Preventive therapy may help reduce cluster attack frequency and severity as well as alleviate accompanying disability [23]. Transitional therapy also plays an important role in the management of CH. This is because it can take several weeks to achieve effects from prophylactics through titration. It serves as a bridge between the initiation of preventive dosing and the proper adjustment of dosage. About the half of patients received transitional therapy and/or preventive therapy with galcanezumab treatment in this study. And patients with episodic cluster headaches who underwent galcanezumab therapy tended to receive a second round of treatment in the early stages of their next cluster bout without transitional therapy. However, there is a limitation in definitively concluding the effectiveness of galcanezumab alone because patients with CH used the multiple treatments combined with galcanezumab treatment in this study. This suggests the complexity of managing CH and emphasizes the importance of tailoring treatment plans to meet individual patient clinical characteristics.

The relatively high percentages (86% and 64%) of “very much better” or “much better” by PGI-I after galcanezumab treatment also supported the effectiveness of galcanezumab treatment in our study. Most patients reported that their CH condition was “very much” or “much better” after galcanezumab treatment. However, PGI-I declined in the second episode, suggesting that patients’ treatment expectations were higher than those in the first episode. The PGI-I scale varies based on the degree of improvement in clinical measures, including reduction in the frequency of CH attack, duration, and severity. According to post-hoc analyses of a phase 3 randomized study, achieving a 43% reduction in attack frequency compared to the baseline was associated with the feeling “much better,” while a 30% reduction corresponded to the feeling “a little better” [24]. This could explain why the percentages “very much” or “much better” of PGI-I and 50% reduction were similar in this study.

In this analysis, no serious adverse drug reaction was noticed with repeated galcanezumab treatment, and the patient did not experience the same adverse drug reaction repeatedly. A previous long-term open-label safety study using galcanezumab has reported that treatment-emergent adverse events (73%) are mostly mild to moderate, with nasopharyngitis being most common [12]. Constipation, pruritus (not associated with the injection site), and vertigo have been identified as adverse drug reactions in the integrated study of patients with migraine receiving galcanezumab for up to 12 months [25]. Therefore, our results align with the previous safety study, demonstrating that repeated exposure to galcanezumab at intervals of 3 to 24 months in patients with CH supports and provides additional safety and tolerability.

Strongest limitation of our study was that the sample size was too small to assess the efficacy of consecutive galcanezumab treatment with various combinations of conventional preventives, different starting dates of galcanezumab treatment, and duration of cluster periods in consecutive cluster bout. Therefore, a selection bias could not be avoided in this study. There is a minor limitation in that details about CH were collected through self-reporting of the participants, which could result in an overestimation or underestimation of the true clinical characteristics of CH attacks.

## Conclusions

In conclusion, it has been confirmed that galcanezumab treatment, as a preventive drug for episodic CH, can lead to varying treatment effects among different episodes of cluster bouts, even within the same patient. And patients with episodic CH who are treated with galcanezumab tend to receive a second treatment in the early stages of next cluster bout without transitional therapy. More extensive and extended trials are necessary to ascertain the galcanezumab’s durability and safety.

## Data Availability

The datasets of the current study are available from the corresponding authors upon reasonable request.

## Abbreviations

CH: Cluster headache
ICHD-3: International Classification of Headache Disorders
PGI-I: Patient Global Impression of improvement
KCHR: CH in the Korean Cluster Headache Registry
BMI: Body mass index
PHQ: Patient Health Questionnaire
GAD: Generalized Anxiety Disorder
EQ-5D: Euro-Quality of Life-5 Dimension
VAS: Visual Analog Scale
IQR: Interquartile range
